# Towards Point-of-Care Heart Failure Diagnostic Platforms: BNP and NT-proBNP Biosensors

**DOI:** 10.3390/s19225003

**Published:** 2019-11-16

**Authors:** Hussein Alawieh, Trishia El Chemaly, Samir Alam, Massoud Khraiche

**Affiliations:** 1 Neural Engineering and Nanobiosensors Group, Biomedical Engineering Program, Maroun Semaan Faculty of Engineering and Architecture, American University of Beirut, Beirut 1107 2020, Lebanon; hma93@mail.aub.edu (H.A.); tce04@mail.aub.edu (T.E.C.); 2 Department of Electrical and Computer Engineering, Maroun Semaan Faculty of Engineering and Architecture, American University of Beirut, Beirut 1107 2020, Lebanon; 3 American University of Beirut Medical Center, Beirut 1107 2020, Lebanon; salam@aub.edu.lb

**Keywords:** heart failure, biomarkers, nanobiosensors, point-of-care-testing, BNP, NT-proBNP

## Abstract

Heart failure is a class of cardiovascular diseases that remains the number one cause of death worldwide with a substantial economic burden of around $18 billion incurred by the healthcare sector in 2017 due to heart failure hospitalization and disease management. Although several laboratory tests have been used for early detection of heart failure, these traditional diagnostic methods still fail to effectively guide clinical decisions, prognosis, and therapy in a timely and cost-effective manner. Recent advances in the design and development of biosensors coupled with the discovery of new clinically relevant cardiac biomarkers are paving the way for breakthroughs in heart failure management. Natriuretic neurohormone peptides, B-type natriuretic peptide (BNP) and N-terminal prohormone of BNP (NT-proBNP), are among the most promising biomarkers for clinical use. Remarkably, they result in an increased diagnostic accuracy of around 80% owing to the strong correlation between their circulating concentrations and different heart failure events. The latter has encouraged research towards developing and optimizing BNP biosensors for rapid and highly sensitive detection in the scope of point-of-care testing. This review sheds light on the advances in BNP and NT-proBNP sensing technologies for point-of-care (POC) applications and highlights the challenges of potential integration of these technologies in the clinic. Optical and electrochemical immunosensors are currently used for BNP sensing. The performance metrics of these biosensors—expressed in terms of sensitivity, selectivity, reproducibility, and other criteria—are compared to those of traditional diagnostic techniques, and the clinical applicability of these biosensors is assessed for their potential integration in point-of-care diagnostic platforms.

## 1. Introduction

Cardiovascular diseases (CVDs)—diseases of the heart and blood vessels—remain the most prevalent causes of death worldwide accounting for 31% of recorded annual mortalities [1]. According to the World Health Organization, cardiovascular diseases include heart failure, rheumatic heart disease, arrhythmia, coronary heart disease, atherosclerosis, atrial fibrillation and cerebrovascular disease [1]. Amongst all the aforementioned forms of CVDs, heart failure is the most debilitating with the highest rates of mortality, morbidity, and healthcare costs. The latter is reported by the American Heart Association (AHA) as amounting to $18 billion direct costs in 2017 [1,2]. Heart failure (HF) describes the condition in which the heart fails to meet the demand of body organs for blood and oxygen; this can be due to the inability of the heart to either fill with or pump out adequate amounts of blood to meet the body’s demand [3]. The clinical symptoms of HF include dyspnea, fatigue, palpitations, and edema [4]. However, such symptoms are nonspecific and are often associated with other diseases such as respiratory infections—especially for elderly patients [5,6,7]. This would eventually undermine diagnosis, prognosis, and disease management and might result in serious health concerns. In addition, while physicians often rely on electrocardiogram (ECG) measurements for diagnosis, these measurements usually fail to delineate HF-related irregularities or warning signs [8,9]. To overcome these limitations to successful patient care and risk stratification, research has shifted focus to getting an in-depth understanding of disease pathogenesis at the cellular or molecular levels by monitoring the concentrations of cardiac biomarkers secreted in the blood [10,11,12]. When it comes to biomarkers in HF, the natriuretic peptides, specifically B-type natriuretic peptide (BNP) and N-terminal prohormone of BNP (NT-proBNP) are considered the gold standard as they stand out with relatively high diagnostic and prognostic relevancy [11,13]. Current BNP testing is confined to conventional immunoassay-based laboratory tests whose results require several hours or even days to be delivered [14]. While incorrect diagnosis of HF could lead to undertreatment, delayed diagnosis of HF can result in false treatment, increased hospital admission, increased cost, and possible permanent heart damage [6,15]. Particularly, in a study done by [16], there was a significant reduction in mortality in those receiving early therapy compared with those whose therapy was delayed by only 35 min. Rapid diagnosis of HF continues to be a challenge; there is a need to reduce the waiting time for patients and allow real-time and accurate monitoring. For that purpose, there has been much interest in the development of diagnostic methods that are rapid enough to be practiced at the bedside, in home-based care and in emergency rooms, and are simple enough to be used by health-care workers that are highly skilled. With that in mind, when it comes to BNP and Pro BNP detection, research has been focused on optical and electrochemical sensors due to their respective high sensitivity and practicality [17]. In this review, we survey BNP detection biosensors for HF diagnosis, and characterize their properties in the scope of point-of-care testing (POCT). The paper starts with an overview of HF pathology followed by a summarized review of the clinically significant cardiac biomarkers for HF diagnosis, prognosis, and therapy. The paper then highlights the advantage of BNP and NT-proBNP in HF management and the need to translate its use from laboratory tests to POCT. For that, the current status of BNP and NT-proBNP biosensors is presented by detailing the comprehensive literature on the proposed optical, electro-chemical, and other BNP biosensing techniques. The reviewed techniques are compared in terms of reproducibility, selectivity, sensitivity, time for delivering response, type of surface functionalization, type of sample used, and other criteria. Based on the latter characteristics of the biosensing techniques, the paper presents key challenges towards achieving POCT-based BNP and NT-proBNP sensing, and it suggests future directions of research.

## 2. Diagnostics in Heart Failure

### 2.1. HF-Specific Cardiac Biomarkers

#### 2.1.1. Cardiac Biomarkers

The physiological changes leading to HF progress in several pathways transforming the pre-risk factors of a normal heart into a symptomatic state of injury [10]. The AHA provides a complementary classification of such stages on the functional, developmental, and symptomatic levels [18]. These stages develop due to a wide variety of heart or valvular diseases that change the behavior of cardiac myocytes causing an imbalance in the size of the heart chambers and the thickness of its walls leading to HF progression [19]. Structural and functional changes in cardiac myocytes elucidate the pathophysiology of HF and are linked to the variation in circulating concentrations of cardiac biomarkers, which trigger cardiac responses and regulatory behavior [11]. HF-related cardiac biomarkers are categorized into seven families—each linked to a specific pathophysiological model of HF; there are markers of myocardial stretch, oxidative stress, neurohormonal stimulation, myocytes injury, inflammation, renal dysfunction, and extracellular remodeling and fibrosis [10,20]. Detection and monitoring such cardiac biomarkers relays critical diagnostic, prognostic, and therapeutic knowledge throughout the complex path from HF risk to chronic HF [11,12,21].

The broad range of biomarkers necessitates the definition of clear assessment schemes for clinical relevance. Authors in [22] highlighted three fundamental criteria for accessing the clinical potential of a new biomarker: (1) it can be assayed in an accurate, high-throughput, rapid, reproducible, and cost-effective manner, (2) it must have a strong and consistent association with the disease and must provide additional information that is not accessible by existing tests or physical examination, (3) it must aid in clinical decision-making on the diagnostic, prognostic, or therapeutic levels. AHA later standardized the critical assessment of cardiovascular risk markers in a scientific statement, which detailed necessary phases of risk marker evaluation before clinical use [22]. Standardized assessment methods are pivotal since statistically significant associations between biomarkers and outcomes do not necessarily imply clinical significance [21].

Over the past few years, several HF related biomarkers were extensively studied and assessed for potential clinical use. Table 1 shows the clinical relevance of BNP compared with other promising cardiac biomarkers [12]. Besides natriuretic peptides, the most prominent biomarkers in clinical practice are cardiac troponins (cTn), C-reactive protein (CRP), myeloperoxidase, tumor necrosis factor (TNF), copeptin, neutrophil gelatinase-associated lipocalin (NGAL), procalcitonin, soluble suppression of tumourigenicity 2 (ST2), and others [23]. Cardiac troponins are indicative of myocytes injury, necrosis, left ventricular (LV) hypertrophy, and systolic dysfunction [10]. cTn and TNF are inflammatory biomarkers highly correlated to the severity of HF [22]. Myeloperoxidase is an oxidative stress biomarker predictive of mortality risk and adverse cardiac events in HF patients. Copeptin, a peptide hormone with antidiuretic and vasoconstrictive properties, is a marker of neurohormonal activation and exhibits elevated levels in HF patients. Copeptin has been extensively studied, and results confirmed its effectiveness in predicting death and its prowess in guiding therapeutic interventions amongst HF patients [10]. NGAL is a polypeptide biomarker for predicting renal injury and failure in chronic and acute HF patients [13]. Procalcitonin is used to distinguish between HF related dyspnea and non-cardiac related dyspnea. Soluble ST2 (sST2) is another biomarker integrating inflammation, fibrosis, and cardiac stress to give valuable prognostic information for risk stratification and management of HF [24,25].

#### 2.1.2. Natriuretic Peptides: BNP and NT-proBNP

While incorporating the above cardiac biomarkers with the traditional diagnostic methods led to some improvement in disease management and risk stratification, reviews of studies on these biomarkers suggest that none can be used as a benchmark for decision-making or as a stand-alone test for guiding clinical judgment in diagnosis, prognosis, and therapy [10,11,12,21]. Remarkably for natriuretic peptides (BNP and Nt-proBNP), there had been substantial evidence that supports their use in diagnosis [26,27,28,29,30,31,32,33,34,35,36,37,38] and prognosis [38,39,40,41,42,43,44,45] with promising studies on the use of natriuretic peptide guided therapy [11,46,47,48,49]. Recently, the American College of Cardiology, the American Heart Association Task Force on Clinical Practice Guidelines, and the Heart Failure Society of America recommended natriuretic peptide biomarker-based screening followed by team-based care as a preventive measure for HF [18]. A major strength of BNP and Nt-proBNP is their high negative predictive value; that is, they are especially useful in the exclusion of HF as a cause of symptoms [46]. Even on the therapeutic level, the use of the more stable and biological inactive Nt-proBNP is emerging with promising outcomes. Other natriuretic peptides are also emerging with added diagnostic significance such as the mid-regional pro atrial natriuretic peptide (MR-proANP), which is increased in response to arterial wall stretch in HF [11,12]. In [50], a study of biomarkers in acute HF (BACH study) showed promise for MR-proANP in HF diagnosis. However, further investigation of its incremental utility is needed [21].

In the past few decades, B-type natriuretic peptides have gained paramount interest as biomarkers in the translational research realm after unveiling their presence in the porcine brain back in 1988 by Sudoh et al. BNP is a biologically active 32 amino-acid peptide neurohormone and is part of the structurally related natriuretic peptides (NPs) family [51]. BNP possesses natriuretic, diuretic, and vasodilative properties. It is formed in the ventricular myocardium as it experiences excessive pressure accumulation due to ventricular dysfunction and is secreted from the cardiac myocytes following pathological and physiological stimuli [8]. The neurohormone acts on maintaining normal blood flow, re-establishing homeostatis, and reducing the built-up pressure by relaxing the vascular smooth muscle, increasing the capillary permeability, increasing glomerular filtration and reducing sodium reabsorption in the ducts [8]. In addition to BNP, there exists NT-proBNP, a biologically inactive 76 amino-acid peptide neurohormone with a mode of action similar to that of BNP. As opposed to BNP, NT-proBNP has a longer biological half-life, and higher in vitro stability, which makes BNP a better candidate for monitoring acute hemodynamic changes in HF patients [8]. Specific to some scenarios as in the cases of HF with preserved LV ejection fraction and asymptotic patients, NT-proBNP possesses better prognostic capabilities and demonstrates better diagnostic accuracy [8]. Over the past years, several experiments were conducted in both outpatient care and urgent care settings to get a comprehensive and quantitative evaluation of the reliability of BNP measurements in HF prognosis, diagnosis and therapy management. It was found that in urgent care and emergency departments BNP blood concentration of 80 pg/mL is an accurate predictor of congestive heart failure with a 95% confidence interval, whereas concentrations less than this are associated with a 98% negative predictive value [52]. The Breathing Not Properly Multinational Study in 2002 is the largest study that showed a convincing utility of BNP in HF diagnostics for more than 1500 patients [27]. The most valuable findings can be summarized as follows: BNP concentrations ≥ 100 pg/mL yielded an average diagnostic accuracy of 83%, BNP values ≤50 pg/mL resulted in a negative predictive value of 96%, a cut-off value of 400 pg/mL indicated presence of HF, a cut-off value of 100 pg/mL indicated absence HF, and the gray zone is between 100–400 pg/mL [13]. Furthermore, it was validated that NT-proBNP exhibited higher levels than BNP in patients admitted to the emergency department with a negative predictive value of 97%, sensitivity of 95%, and specificity of 68% [13]. Upon this study, BNP began to catch on clinically. In 10 years following the study, BNP moved from being just a biomarker to a useful tool in cardiology, specifically in outpatient clinics, screening, and risk prediction [53]. The studies leading up to the takeoff of BNP include the PRIDE (N-Terminal Pro-BNP Investigation of Dyspnea in the Emergency Department), ADHERE (Acute Decompensated Heart Failure National Registry), PROTECT (Use of NT-proBNP Testing to Guide Heart Failure Therapy in the Outpatient Setting), HOPE (Heart Outcomes Prevention Evaluation) studies, and more [15,54,55]. The coalition of the results of more than six relevant studies leads to a conclusion that introducing BNP and NT-proBNP to clinical judgment resulted in an enhanced HF diagnostic accuracy of around 71% to 84% [52]. Moreover, in adjunct to their role in diagnosis and prognosis, BNP and NT-proBNP can be potentially used in directing HF pharmacological strategies and medications. When used as part of the routine assessment of dyspnea patients, they have contributed to the decrease in treatment costs and length of hospitalization [15]. Finally, with all this presented, it can be confirmed that BNP and NT-proBNP outperform conventional biomarkers with high concentrations being directly correlated to acute and chronic HF as well as mortality rates in patients [8].

### 2.2. Traditional Immunoassay BNP Tests

In an attempt to quantify the level of cardiac biomarkers, including BNP, different immunoassays have been developed.

#### 2.2.1. Radioimmunoassays

The first immunoassays used for BNP detection areradioimmunoassays (RIAs), which make use of a competitive behavior between radioactively labeled antigens and target antigens in binding to available immobilized antibodies [56]. In such case, the generated radioactive signal is inversely related to the concentration of target antigens. However, these methods suffer from lack of specificity, lack of automation, and increased health hazards due to the radioactivity employed in their design.

#### 2.2.2. Fluorescent-Based Immunoassays

Another kind of immunoassays is fluorescent-based. It uses fluorescent probes conjugated to the antibodies. The latter requires costly instruments, trained personnel, and fluorescent labelling [57].

#### 2.2.3. ELISA

Another popular assay is the enzyme-linked immunosorbent assay (ELISA). In ELISA, the antibodies are linked to an enzyme specific to a reaction of a substrate. After the antibodies are allowed to bind to the antigen of interest (BNP), the substrate is introduced, and its catalyzed reaction produces a color change that helps quantify BNP. The limitations of ELISA include the need for a large sample volume, long detection time of about four hours, and the cost of instruments. To add, compared to other types of assays, ELISA is not sensitive enough to detect too small BNP levels.

#### 2.2.4. ECLIA

A fourth type of immunoassays is the electrochemiluminescent immunoassay (ECLIA) which quantifies the emission of photons by the relaxation of electrons from high energy levels. It is characterized by sensitivity, specificity, possibility for miniaturization, and automated operation. However, as previous immunoassays, it still requires costly and large analytical instruments, operating personnel, and delayed response time [58].

**Figure 1:**
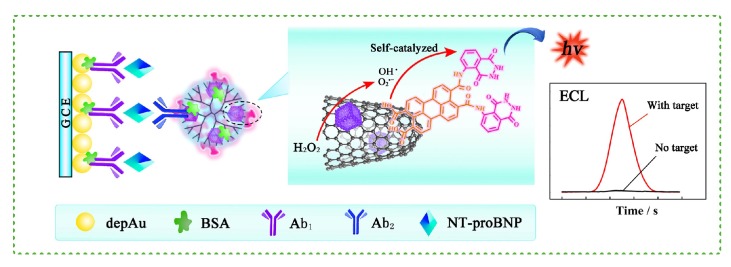
Schematic of a sandwich-type BNP immunoassay used with an ECL immunosensor [60]. Glassy carbon electrodes, modified with Au nanoflowers, are functionalized with primary antibodies. Secondary antibodies are conjugated to single wall carbon nanohorns linked to bimettalic nanocubes and loaded with a novel self-catalyzed luminescence emitter.

All four immunoassays also fail to achieve real-time monitoring of the patient’s cycles throughout the day. In the search for a rapid, low-cost, sensitive, real-time approach for the detection of BNP at low concentrations and in emergency rooms, current biosensors involve the use of immunoassays but aim at addressing their limitations [6]. Sandwich type immunoassays are commonly used with biosensors. They consist of primary antibodies that are immobilized on a surface, the antigen that binds to the primary antibodies, and secondary antibodies that bind to remaining free epitopes of the antigen, thus forming a sandwich [59]. Figure 1 illustrates the schematic of a sandwich type immunoassay that is used within the design of an electrochemiluminescent (ECL) BNP immunosensor [60].

### 2.3. Need for Point of Care Testing

Currently, testing for BNP is still confined to the medical laboratory where conventional immunoassay-based tests are used. Results may require several hours or even days to be delivered while medical care continues without the required diagnostic information. However, immediate diagnosis of heart failure is essential to ensure early and appropriate therapy. Delayed diagnosis of HF can be linked with inappropriate treatment, increased health-care costs, longer hospitalization, and more frequent visits to emergency rooms [6]. Particularly, the authors in [61] correlate the delay in delivering therapy to the increased rate of mortality and increased duration of stay in the hospital. In addition, authors in [61] also correlate HF complications with time-to-treatment: patients who received prompt treatment had shorter hospital stays and a greater probability of being asymptomatic at discharge while patients who had delayed therapy experienced more adverse impact. All in all, routine and prompt BNP testing is essential to reduce time-to-treatment and thus improve therapeutic outcomes. On that account, POCT is required for bedside testing or in the emergency room, for real-time accurate monitoring.

## 3. Biosensors for BNP Detection

**Figure 2:**
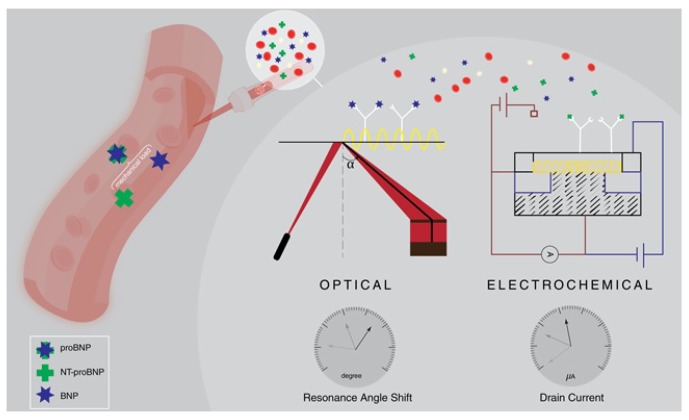
Electrochemical and optical nanobiosensing of BNP and Nt-proBNP.

To address the need for POC for rapid diagnostic methods, BNP biosensors are being developed. Biosensors transform an immunoassay event into a detectable signal representing the level of a biomarker, particularly BNP in this review. The main focus in biosensors is on optimizing and amplifying the transduced signal relating to the concentration of the analyte using different detection paradigms. This review covers advances in optical and electrochemical sensors towards point-of-care applications. Table 2 presents a comprehensive comparative analysis for the studied optical and electrochemical biosensors. An illustrative example on optical (SPR-based) and electrochemical (potentiometric) nano-biosensing is depicted in Figure 2.

### 3.1. Optical Immunosensors

Optical sensing aims at detecting an immunoassay recognition event where the immunosensor could either be optical intensity based (fluorescent or luminescent), surface plasmon based, or Raman spectroscopy based [57]. In general, optical sensors possess impressive sensitivity that reaches down to ng/mL and pg/mL. This section of the review covers recent optical immunosensors for BNP detection.

#### 3.1.1. Surface Plasmon Based Biosensors

Surface plasmon resonance (SPR) is essentially used in biosensing due to the sensitivity of the surface plasmons’ resonance to the interface’s dielectric [62]. This sensing method is rapid, simple, low-power, and label-free and allows real-time monitoring, which is required for POC. However, it lacks sensitivity for small molecules such as BNP (tens of nanomolars) compared to conventional immunoassays and faces nonspecific adsorption in complex solutions such as serum [63,64]. In that context, several works have been proposed in the aim of increasing SPR sensitivity by signal amplification, specifically by conjugating nanoparticles to secondary antibodies in a surface sandwich assay or by indirect quantification of BNP.

Authors in [65] presented a method for increasing the sensitivity of SPR-based BNP detection from ng to pg using 50 nm beads for SPR signal amplification. Initially, a sandwich-type immunoassay was prepared using primary and secondary anti-BNP Abs. In that initial setup, the detected SPR angle increased with the BNP concentration, especially after the introduction of the secondary Abs. However, the lower limit of detection was about 10 ng/mL of BNP, and no angle shift was monitored at lower concentrations. To increase the sensitivity, streptavidin-labeled nanobeads that are larger in volume than the secondary Abs and could bind to them by the biotin-streptavidin interaction were added to the setup. Abs and nanobeads were alternately introduced after the secondary Abs to form four layers. This helped achieve a clinically significant detection limit of 25 pg/mL. Unfortunately, though, this sensitivity was not achieved when using human plasma due to nonspecific adsorption, the prevention of which is difficult [65].

Within the same trend of improving SPR-based measurement by signal amplification, an approach which combines DNA aptamer functionalized gold surface and secondary Ab functionalized gold 50 nm edge nanocubes in a sandwich-type immunoassay was introduced [64]. The approach required an hour of incubation in BNP and was able to achieve a detection limit of 1 aM. By controlling concentrations of the secondary probe, a dynamic range of 1 aM to 500 nM (11 orders of magnitude) was achieved. Thus, a wide dynamic range was covered without compromising the detection limit. The immunosensor was then tested with undiluted human serum to account for nonspecific adsorption where monitoring of BNP was possible in <10 aM range and in the 60–100 aM range with 0.16 and 0.09 nM of the secondary probe, respectively. However, the SPR response was still lower due to nonspecific adsorption [64].

Another trend is the development of lab-on-a-chip small, light, portable, and rapid systems which can be employed in POC [63]. One proposed sensor in that context is a T-shaped microfluidic system with a portable SPR system. It increased the sensitivity of an SPR-based system by the quantification of an enzymatic product, to indirectly quantify BNP. The T-shaped system was made of two perpendicular gold thin film microchannels A and B. A was modified with BNP by an EDC/NHS mediated reaction. If a solution of BNP and antiBNP-AChE (AChE: acetylcholine) are introduced through A, BNP and antiBNP-AChE undergo an immunoreaction, and the unreacted antiBNP-AChE is collected. After rinsing with a buffer, acetylthiocholine, an AChE substrate, is introduced through channel B. The enzymatic reaction between the trapped AChE and the introduced acetylthiocholine forms thiocholine which accumulates on B. This thiocholine monolayer causes an SPR angle shift that could be related to the concentration of BNP. Notably, if BNP concentration is increased, the trapped AChE is decreased, the thiocholine concentration is decreased, and the SPR angle shift is decreased. The proposed method reached a detection limit of 5 pg/mL of BNP and a wide dynamic range of 5 pg/mL to 100 ng/mL in a BNP solution. It was then suppressed for nonspecific adsorption and tested with human serum, and the results confirmed the previous ones within 10 pg/mL to 100 ng/mL. In addition, the whole assay was completed in 30 min, which is acceptable but could still be improved by automating the manual process and increasing the area of BNP immobilization [63].

**Figure 3:**
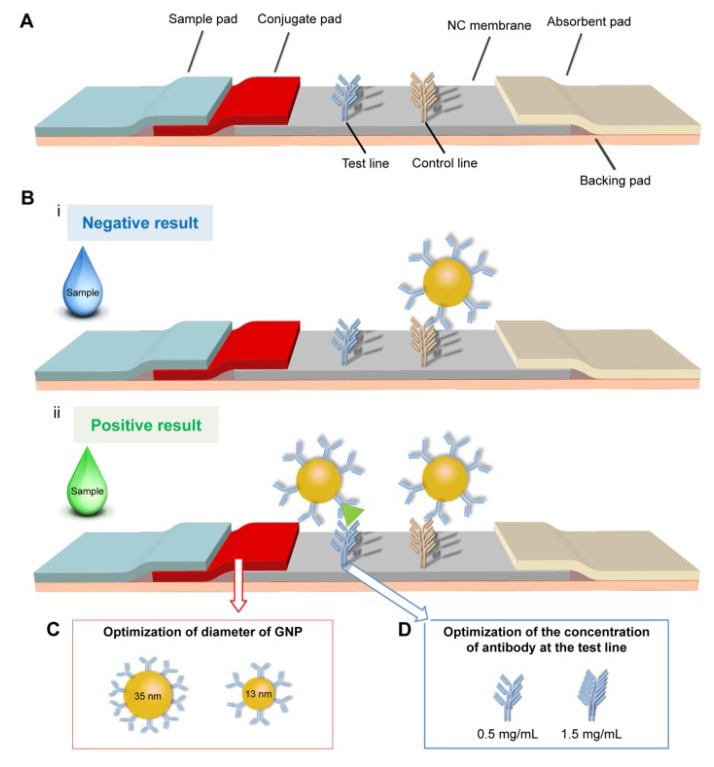
Schematic of the optimization of a LFIA for the point-of-care detection of BNP [66]. (**A**) a thin conjugate pad added to get a complete release of GNP-Ab; (**B**) LFIA principle; (**C**) adjustment of GNP diameter to increase the signal; (**D**) adjustment of the concentration of the test line to increase the signal.

More recently, an improved lateral flow immunoassay (LFIA) was developed for POC applications. The proposed method relies on an on-strip sandwich reaction between BNPs and their Abs. Particularly, the formation of the complexes at the test line produces a qualitatively detectable signal that could also be quantified based on the optical density [66]. The latter method is depicted in Figure 3. Although lateral flow immunoassays suffer from poor sensitivity, they are easy to use and affordable. To make use of these properties, the authors in [66] aim to increase the sensitivity of the assay by improving GNP-Ab conjugate conditions, the diameter of the GNPs used, the concentration of the Abs on the test line, and the structure of the test strip. Specifically, they use particles of diameter 35 nm and a concentration of 1.5 mg/mL for the Abs. Subsequently, BNP was detected at a limit of 0.1 ng/mL within 10 to 15 min, with an improved sensitivity (15 fold) over the conventional lateral flow assay. In addition, the system preserved its sensitivity when tested with human serum and showed good specificity in the presence of interfering cardiac biomarkers (CRP, Myo, and cTnI), proving the possibility of it being used for POC detection of HF with the naked eye.

SPR-based sensors are rapid, simple, low-power, and label-free which makes them cheap. They also have in situ capabilities, high spatial resolution and allow real-time monitoring. They can identify specific and nonspecific adsorption, which should be solved by purifying the samples or suppression. This issue is to be further addressed for POC applications. To add, bulky detection instruments are needed, especially for the Kritschmann configuration, but research regarding this issue is being conducted where portable and disposable SPR sensors have been proposed. Real-time monitoring in vivo has also been achieved through direct insertion of an SPR sensor into a vein, but not for BNP detection [57].

#### 3.1.2. Optical Intensity Based Biosensors

The output of an optical intensity based biosensor is either a fluorescent or luminescent signal. This section of the review covers recent electrochemiluminescence (ECL) and fluorescent immunosensors. It also includes certain efforts to improve the signals derived from these sensors by the use of gold and magnetic nanoparticles.

Very recently, an ECL immunosensor with good selectivity and sensitivity for BNP was introduced [60]. A novel self-catalyzed luminescence emitter was proposed by conjugating 3, 4, 9, 10-Perylenetetracarboxylic acid (PTCA) with luminol into PTC-Lu. This is because PTCA catalyzes the oxidation of luminol by H_2_O_2_ into a highly energized product that emits the extra energy as a light photon. In parallel, a nanohybrid was prepared by conjugating single wall carbon nanohorns (SWCNHs) to PdCu bimettalic nanocubes into PdCu@SWCNHs. SWCNHS were used as nanocarriers due to their low toxicity, excellent conductivity and biocompatibility in addition to their electrocatalytic behavior, which can further amplify the ECL signal. The nanocarriers were loaded with PTC-Lu to form PTC-Lu/PdCu@SWCNHs. Glassy carbon electrodes, modified with Au nanoflowers (AuNF) due to their large surface area and goodconductivity, were functionalized with NT-proBNP Ab1. The electrodes were then incubated in a solution of NT-proBNP for 45 min followed by the binding of the PTC-Lu/PdCu@SWCNHs-Ab2 bioconjugate to form a sandwich immunoassay (as in Figure 1) [60]. The ECL response of the sensor to different NT-proBNP concentrations was investigated where the intensity increased with increasing concentrations in a linear relationship to the logarithm of the concentrations at a detection limit of 0.05 pg/mL, with a dynamic range of 0.1 pg/mL to 25 ng/mL. To confirm specificity, the immunosensor ECL response was higher to NT-proBNP at 0.1 ng/mL than to other proteins (AFP, Col IV, and PSA) at 5 ng/mL and was also conserved in a mixture of these proteins. Stability was also confirmed via a 1.9% standard deviation of ECL intensity over 12 cycles. Reproducibility was confirmed with a 3.48% intra-assay precision and a 2.73% inter-assay precision. Finally, a comparison of the sensor’s performance with undiluted human serum samples to that of a clinical immunoassay gave a relative deviation of 5.0–6.0% [60]. Although ECL methods have high specificity and sensitivity, they may require large instruments and trained personnel. In parallel, immunochromatographic assays are simple and rapid. On this basis, a method to quantify NT-proBNP in human serum, plasma, and whole blood with no significant variation was proposed. It involves the use of fluorescent-streptavidin which possesses high quantum yield and good light absorption. The test strip had several pads: one with biotin labeled anti-NT-proBNP, another with the streptavidin modified fluorescent protein. As the sample flows in, a complex of NT-proBNP-anti-NT-proBNP antibody-biotin-fluorescent protein forms. The complex then immobilizes on a nitrocellulose membrane that is functionalized with anti-NT-proBNP. The test strip was used with 10 μL samples and inserted into a fluorescence detector with reaction time of 10 min, the minimum time needed to establish equilibrium intensity. The test precision was evaluated based on the coefficient of variation among inter and intra assays, which proved to be less than 10%. The test strip was also compared to chemiluminescence immunoassay CLIA, attaining a correlation coefficient of 0.978. A wide linear range of 200 pg/mL to 26,000 pg/mL and a detection limit of 47 pg/mL were obtained. The authors concluded that, due to its precision, accuracy, sensitivity, and rapidity, the proposed test could be developed into a platform or commercial kit to be used for POC and emergency applications [58].

Another proposed method aimed to improve the sensitivity of fluorophore-mediated biosensors by enhancing fluorescence. Nanogold particles (NGP) can enhance fluorescence by transferring free electrons when placed at a particular distance from fluorophores and reducing self-quenching, and so can organic solvents by different means. In this context, researchers combined NGP-SAMs and solvents into nanogold particle reagent (NGPR) [67]. Cardiac marker sensors including BNP sensors were prepared by sandwich immunoassay. The distance between the NGPs and fluorophore conjugated second Abs was adjusted by changing the thickness of the SAM (1, 2, or 3 nm) in NGP-SAMs while their concentrations were kept constant. Experiments were first conducted with protein C sensors where 2 nm SAMs gave the best enhancement, reaching 215% increase in fluorescence, meaning 1 nm was too close and 3 nm was too far. The NGP size was also tested for enhancement where 10 nm NGPs gave the best enhancement among 2, 5, and 10 nm. However, the best overall enhancer was 5 nm NGPs with 2 nm SAMs. The use of NGPRs also proved to show additive enhancement, showing a 10 times stronger signal. Then, NGPR enhancement was evaluated with a fiber-optic BNP sensor of range 26–260 pM with 5 min incubation. It showed that the signal was amplified 2.5 times.

Some proposed methods have targeted signal enhancement of NGP-based lateral flow assays and could be translated to BNP and NT-proBNP biosensors. A one-step signal amplification method using oligonucleotide-linked NGP aggregates was used to enhance the sensitivity in nucleic acid lateral flow assays and improve detection limit by 2.5-fold [68]. NGPs could also be conjugated to polyclonal secondary antibodies, thus allowing multiple binding and enhancing the detected signal [69]. A third approach addresses instability issues with the use of NGPs by modifying their surfaces with polyethylene glycol that is also known to reduce non-selective adsorption [70].

The previously used nanoparticles are known for large surface area, reduced size, biocompatibility, and stability. Magnetic nanoparticles in particular are transportable and possess unique physicochemical properties that make them attractive for POC. A proposed work combined micro-magnetic particles (MMPs) with sandwich-type ELISA, making use of their mobility to shorten reaction time and reagent volume. Horseradish peroxidase (HRP) was also used for signal amplification through its catalyzing action on a chromogenic substrate into a detectable colored product. For the sandwich-type immunoassay, MMPs were conjugated to capture Abs, forming magnetic probes that could sandwich BNP along with HRP-labeled Abs. The proposed sensing method consisted of two incubation reaction steps and one wash step where the complexes were magnetically separated, giving an overall detection time of 30 min. The influence of some important parameters on detection was then studied. Particularly, the use of MMPs allowed reducing negative signals, thus improving sensitivity. The combined signal amplification of MMPs and HRP allowed reducing the detection limit to 10 pg/mL of BNP. As a part of verification, the test was finally used on whole blood samples, showing agreement with clinically available methods. Overall, the proposed method improved sensitivity, rapidity, and simplicity of conventional ELISA and reduced the technical proficiency required for operation [59].

Another optical, fluorescence based test was proposed for the quantification of NT-proBNP within 30 min at room temperature [71]. Ab-DNA conjugates (cAB-DNA) were used for capture of NT-proBNP, and fluorobead-labeled Abs (FB-dAB), were used for detection. The test procedure involved 11 min incubation of 10 μL clinical plasma samples with the capture Abs followed by the addition of the fluorescent detection Abs to form a FB-dAB-NT-proBNP-cAb-DNA complex. The mixture containing the complex was then loaded on test strips where glass fiber membrane platforms immobilized with oligonucleotides allowed the capture of the complex via DNA hybridization. Such test strips could be scanned by a biometric reader. The limit of detection was then quantified as 3.7 pg/mL. The test also preserved linearity in tests with human plasma samples, spiked or not spiked with NT-proBNP then serially diluted with analyte-free plasma to obtain desired concentrations. A coefficient of variation of less than 10% proved the test’s accuracy within a detection range of 7–600 pg/mL. The test’s specificity was also evaluated by spiking the plasma with bilirubin, intra-lipid, biotin, and hemoglobin that could possibly cause interference. The correlation coefficient values for the response in the absence and presence of these agents were more than 0.98, indicating very low interference and high specificity to NT-proBNP [71].

One of the recently developed fluorescent sensors is a household prognosis platform with a smartphone-based reader and analysis app [72]. Two biomarkers, BNP and ST2, were simultaneously quantified using dual-color core-shell upconversion nanoparticles as probes. The lateral flow strip reached detection limits of 5 pg/mL and 1 ng/mL and detected minimal concentrations of 17.46 pg/mL and 29.92 ng/mL in clinical samples, for BNP and ST2, respectively. Another up-converting phosphor technology-based lateral flow assay was developed to quantify NT-proBNP in human plasma and performed consistently with the Roche Elecsys assay, reaching a limit of detection of 116 ng/L with a coefficient of variation less than 15% [73]. A lateral flow immunoassay for NT-proBNP using antibody-labeled quantum dots was also developed and used monoclonal antibodies, engineered with high specificity to the tail of NT-proBNP. The lateral flow immunoassay showed a linear response range and repeatability with pooled variance of 8.8% across concentrations of NT-proBNP [74].

In optical intensity based methods, signal production and acquisition require simpler and less expensive instruments, as well as less excitation than SPR. However, they require complicated labeling and rely on indirect indicator based signal schemes.

While this review outlines a list of surface plasmon based and optical intensity based biosensors, Raman spectroscopy biosensors, such as surface enhanced Raman spectroscopy (SERS) nanotags-based lateral flow assays, have also been developed for the detection of cardiac biomarkers. Raman reporter molecules are conjugated to gold nanoparticles, which are linked to target-specific antibodies. For instance, a lateral flow assay based on Raman dyes encoded core-shell SERS nanotags was developed for the detection of three cardiac biomarkers on a single test line [75].

### 3.2. Electrochemical

With increased miniaturization, considerably high sensitivity and specificity, and a wide variety of biocompatible methods of fabrication and surface functionalization, electrochemical transduction has emerged as one of the most promising techniques for the development of biosensors [17]. Especially for CVD diagnosis and prognosis, immuno-based electrochemical biosensors combine the high specificity of traditional immunoassay methods with the low-cost, simplicity, speed, real-time response, and reliability of modern biosensors [76]. Using immuno-reagents as bio-receptors, the transduced signal either comes from the direct response caused by the specific binding of the target analyte to the receptor antibody or from the indirect response caused by a byproduct of an analyte reaction catalyzed by an enzyme conjugated to the antibody [76]. Such responses can be expressed in terms of charge, pH, current, or impedance changes that correlate to the change of analyte concentration. This gives rise to the classification of electrochemical biosensors into the following categories: potentiometric, amperometric, impedance-based, and conductometric. The incorporation of nano-scale materials in each of the aforementioned categories provides a platform for developing ultra-sensitive sensors with increased surface area, higher reactivity, and amplified response [77]. With such potential improvements, electrochemical biosensors can lead to breakthroughs towards HF POCT.

#### 3.2.1. Potentiometric

Potentiometric immuno-sensors measure the current change of an ion-sensitive field effect transistor (ISFET) in response to the change in its gate surface charge. This charge is logarithmically related to the concentration of the analyte that binds to the immuno-functionalized gate. The well-established FET technology with its impressive miniaturization and practicality makes potentiometric sensors a convenient option for POCT. Work in [78] presented an improvement to the conventional FET design by adding a uniform and high yield two-dimensional (2D) polyaniline (PANI) layer to the gate surface. The extremely rough surface morphology introduced by the PANI layer—and verified by scanning electron microscopy (SEM) and atomic force microscopy (AFM) images—led to an increase of 46% in surface area compared to that of a flat surface [78]. This eventually resulted to a higher surface-area-to-volume ratio; thus, it provided more binding sites which enabled better molecular adsorption and higher reactivity. The measured current response, obtained within one minute in most experiments, showed a linear response within the range of 50–200 pg/mL with an 80% confidence across 20 tested devices. The specificity of the sensor was also tested using non-specific cardiac biomarkers (cTni, CK-MB, and Myo), which showed an insignificant deviation (within ±5%) from the reference BNP response. Authors in [77] also investigated the deviation in the response due to changes in Debye length, which is the distance from the PANI surface after which charge changes would be screened out and would not contribute to current changes in the FET. Debye length is inversely proportional to the square root of the solution’s ionic strength. Such investigation was carried out using different buffer dilutions with the same BNP concentration and aimed at modeling the variations in responses for different serum samples that often possess different ionic strengths. Results proved that sensitivity increased greatly with the decrease of the ionic strength of the solution—or equivalently the increase in Debye length [78].

**Figure 4:**
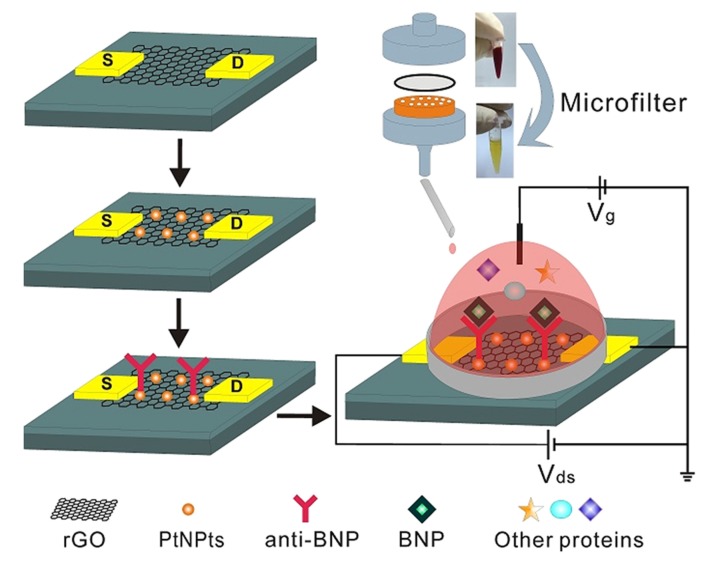
Schematic of a BNP immunoassay on a rGO FET decorated with PtNPsfor signal amplification. The biosensor employs a custom-made microfilter and polycarbonate membranes to allow measurement in whole blood by removing blood cells [79].

Another enhanced potentiometric immuno-sensor design was proposed in [79] to detect BNP in whole blood. Platinum nanoparticles (PtNPs) were deposited on a reduced graphene oxide (rGO) FET in order to amplify the electrical signal by facilitating electron transfer and providing higher surface area. SEM, transmission electron microscopy (TEM), energy dispersion spectrum (EDS), and X-ray photoelectron spectroscopy (XPS) were used to characterize the surface and observe the morphology of PtNPs. Upon adding different concentrations of BNP, I–V characteristics were obtained within 10 s. Linear responses were obtained in the dynamic range of 100 fM–1 nM with reproducibility across two devices [79]. The sensor was stable over time as it retained 85% of its original response value after one week. Its response could also be regenerated after three cycles of cleaning using a high ionic solution (NaCl, 1M) that dissociates the binding between BNP and anti-BNP for re-use. The sensor also proved to be selective against non-specific proteins including Bovine Serum Albumin (BSA), D-Dimer, and Human Albumin solution (HAS) with mean response deviation less than 13%. To allow measurement in whole blood, the sensor was coupled with a micro-filter system as depicted in Figure 4; this required an incubation time of 30 min before getting the response. Tests on human blood samples with BNP concentrations between 50 to 200 nM demonstrated the sensors ability to detect BNP from whole blood [79].

**Figure 5:**
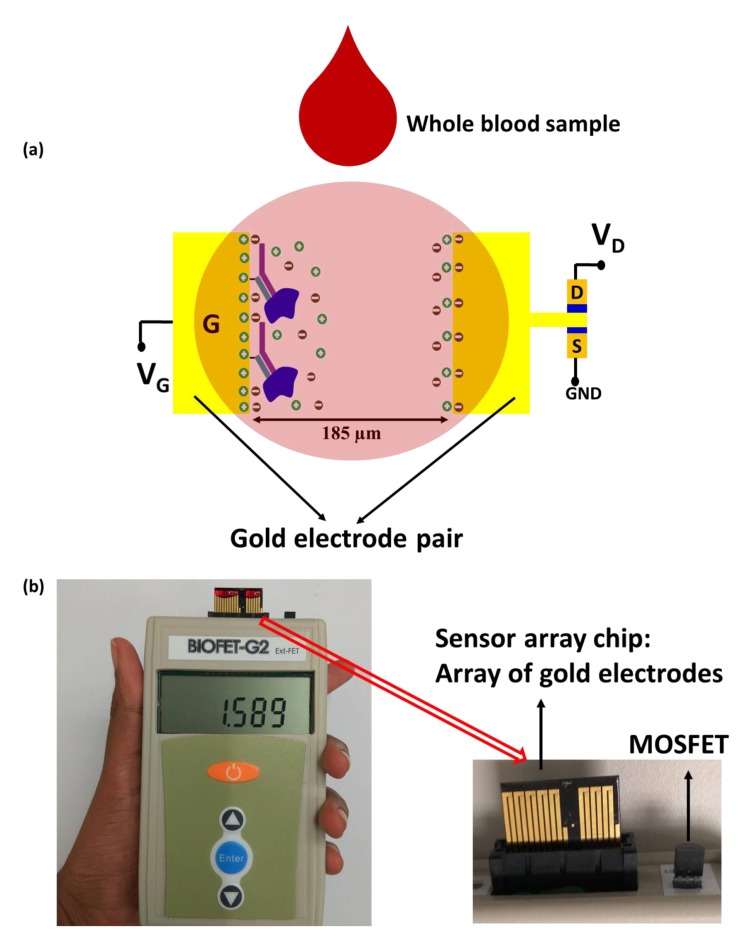
Extended gate electric double layer (EDL) FET biosensor [80]. (**a**) schematic illustration of the biosensor; (**b**) real image of the hand-held measurement system integrating the sensor array chip.

As handheld devices are desired for POC, a handheld biosensor [80] was able to test for BNP in a single drop of whole blood in 5 min. As the blood is not pretreated, charge screening is eliminated, thus enabling detection beyond the Debye length. The proposed system involves an extended gate design of electrical double layer field effect transistor where the gate is linked to a pair of gold electrodes as illustrated in Figure 5. One of the electrodes is functionalized with a BNP receptor so when BNP binds to the receptors, the double layer capacitance and the resulting drain current change. However, the authors evaluate current gain instead of drain current. The system is highly sensitive, with a range of 0 to 1000 pg/mL, preserving its properties even in the presence of background proteins and blood cells.

#### 3.2.2. Amperometric

Their successful implementation in glucose sensing, amperometric biosensors have been highly developed and used in commercial products [17]. These sensors measure the current change due to redox reactions and electron transfer on their working electrodes. A major advantage of amperometric sensors is that, unlike potentiometric sensors, they exhibit a linear current response in relation to the analyte concentration. Their versatility and linearity give them an added advantage towards POCT. However, due to the non-electrochemical activity of antigens and Abs in immuno-sensing applications like BNP detection, there is a need for an indirect electrochemical immunoassay to provide electroactive species to transfer electrons in redox reactions. To this purpose, immobilized Abs are labeled with an electroactive substance or an enzyme so that the concentration of the analyte can be indirectly measured by measuring the current resulting from the reaction of the electroactive species or the enzyme-catalyzed reaction of the analyte. Redox mediators that undergo intermediate redox reactions are often used to enhance electron transfer at the electrode. In addition, the incorporation of nanomaterials can further enhance the performance of amperometric biosensors.

In [81], the use of magnetic nanoparticles allowed the sensor to be regeneration-free where the functionalized magnetic particles can be washed off by simply releasing them, unlike normal immunosensors that could only be regenerated for a few times by chemical conditioning. Magnetic nanoparticles modified with avidin were functionalized with biotinylated anti-NT-proBNP Fab monoclonal antibodies and were attracted to the sensor surface by means of a permanent magnet. Detection of NT-proBNP was based on the change of the current response for cases with and without antigen-Ab recognition on the magnetic nanoparticles. The resulting signal, recorded by cyclic voltammetry (CV), decreased as the concentration of NT-proBNP was increased, giving a linear response within the dynamic range 0.04 and 2.5 ng/mL and a limit of detection (LOD) equal to 0.03 ng/mL. For validation, the immunosensor was tested with 100 human serum samples. The comparison of the result to CLIA gave a high correlation coefficient with only four false alarms, proving possibility for clinical application. This work was further developed by the authors in [82] by adding: Prussian Blue nanomaterials (PPN) as electrochemical mediators and Platinum as a catalyst for hydrogen peroxide oxidation. A sandwich-type immunoassay was formed using PNN-labeled detection antibodies and biotinylated capture antibodies (Bio-Abs) that bind to avidin functional magnetic nanoparticles. The surface and the nanoparticles were characterized using SEM, TEM, and XPS to empirically optimize functionalization conditions. High sensitivity was achieved, within an incubation time of 18 min, with linear dynamic ranges (between 0.005–1.67 ng/mL and 1.67–4 ng/mL) and an LOD of 0.003 ng/mL. The response was also stable for periodic tests (every three days) over a month showing a standard deviation of 2.3% only. The stability was also validated by the low standard deviation error of 2.1% after 50 cycles of sensor use. The selectivity of the sensor was tested using three incubating solutions: healthy serum, Phosphate-buffered Saline (PBS) with non-specific interference (BSA, Tween 20, dopamine and vitamin C), and PBS without interference. Only 4.3% difference was observed between the responses in the three solutions. The sensors was also used against whole blood samples and showed BNP detection results similar to the chemiluminescence immunoassay (CLIA).

Work in [83] presents another approach for using a sandwich-type immunoassay in designing a disposable amperometric sensor. Peroxidase-labeled detector Abs were used and capture Abs were immobilized on gold nanoparticles grafted on screen-printed carbon electrode by means of aryl diazonium salt chemistries. Electrode surface morphology was characterized by AFM, energy-dispersive X-ray spectroscopy (EDX), and SEM and its interfacial properties were investigated using electrochemical impedance spectroscopy (EIS) measurements. The sensor response, obtained by CV measurements after 45 min of incubation, showed a linear response in the range between 0.014 and 15 ng/mL with an LOD of 4 pg/mL [83]. Responses had a storage stability of at least 25 days and high reproducibility with insignificant deviation across five sensors. The selectivity of the sensor was validated by tests using CRP, cTnI, cTNT, L(a), TNF alpha, Interleukin (IL)-8, NTproBNP, and receptor tyrosine kinase AXL biomarkers.

#### 3.2.3. Impedance-Based

Impedance-based electrochemical biosensors make use of an equivalent circuit model for the sensor interface. This model comprises resistive, capacitive, and inductive components that give rise to a total characteristic impedance. Electrochemical Impedance spectroscopy (EIS) is an efficient method to monitor the changes of the interface impedance when the analyte binds to the functionalized surface. In order to amplify the change in impedance, authors in [84] presented a silicon nanosensor that integrates a nanoporous alumina membranes with the sensor’s microfabricated chip. Individual pores were converted into nanowells with picoliter volume that isolates monoclonal antibodies. This would mimic the in vivo spatial confinement of proteins and would enhance their stability and accelerate their folding preventing their potential denaturing as in the case of aggregated in vitro studies [84]. Anti-BNP Abs were immobilized inside the nanowells through covalent bonding to the amine ends of thiol groups that were functionalized on the surface. EIS measurements upon introducing BNP concentrations showed a very high sensitivity of 1 ag/mL and a dynamic range of 1 ag/mL to 10 μg/mL with a standard mean error less than 1%. Measurements were obtained after a 15 min incubation period and were averaged over multiple sensing sites—each with approximately a quarter million nanowells—to reduce variability and increase signal-to-noise ratio. With such ultrasensitive detection, impedance-based biosensors can compete with traditional immunoassays while still resulting in an enhanced practicality for POCT. In the context of POC, a hand-held device [85] was able to combine a lab-on-a-chip module with interdigitated circular capacitive electrodes for label-free detection of BNP. Due to the electron exchange between Ab and Ag, the change in the dielectric constant creates a measurable change in the capacitance. The device then uses saved data to quantify the presence of the biomarker. It is able to report results in less than 30 min human serum.

#### 3.2.4. Conductometric

The working principal of conductometric immuno-sensors is based on measuring the change in conductance due to the change of the type and number of ion species in the solution after the binding of the analyte to the immobilized antibodies. Measurements are done in the presence of an applied electric field. In [86], authors present a single site-specific PANI nanowire biosensor for BNP and other cardiac biomarkers detection. Authors used SEM, fluorescently labeled antibodies, and Raman spectroscopy characterize the surface and optimize the functionalization conditions to yield a cost-efficient design that meets the required sensing performance [86]. A key factor in surface functionalization is the concentration of the immobilized Abs. An insufficiently functionalized surface is undesirable since it would lead to an insufficient conduction change. Similarly, an excessively functionalized surface is undesirable since it increases the risk of crosslink formation between carboxyl and amine groups of the Abs. This reduces the number of available binding sites and thus decreases sensitivity. After optimizing the conditions and parameters for functionalization, an increased sensitivity was achieved over the dynamic range of 50 fg/mL to 3 ng/mL with a standard deviation less than 15% [86]. In addition, the biosensor showed a very high specificity when tested for non-specific interactions of BSA and other cardiac biomarkers. Conductometric sensing was used to measure the sensor response, which was available within few minutes from introducing the sample under test. Notably, the large influence of the solution’s ionic strength and the nonspecific adsorption effects on conductometric biosensors impeded their further development.

## 4. Limitations for POCT

The advantage of POCT is that they can be done by health care workers without technical training [6]. This allows test results to be available to patients and care providers rapidly, at the bedside or in emergency rooms. Given the impact of timely testing on prognosis and the significant diagnostic relevance of the BNP family for HF, there is a need for a POCT for BNP screening. The work discussed here shows great promise toward this goal, but there remain several challenges to overcome before clinical translation of BNP and NT Pro-BNP biosensors to POCT platforms. That being said, there are many challenges for the translation of biosensor technologies to clinical POCT platforms, and it lies in the need to satisfy the size, cost, speed, and sensitivity requirements. Optical sensing methods require bulky and expensive detection instruments, particularly SPR instruments for the Kritschmann configuration. Portable and robust SPR devices are needed but miniaturization remains a big problem. At this point, optical methods may need further technical development to allow miniaturization and lower cost detection. On the other hand, although have an advantage when it comes to lower cost, electrochemical sensing suffers from several issues that impact sensitivity including; ion screening, nonlinear response, the need for electron mediators that undergo intermediate redox reactions to enhance electron transfer, the capacitance of the double layer at the electrode surface, and the dependence on the ionic strength of the solution. Another issue that limits the sensitivity of all sensing techniques is the nonspecific binding (NSB) that results from the binding of labeled secondary antibodies to non-antigen sites on the sensor. The latter contributes to the generation of false signals that degrade sensitivity. It is also worth noting that several approaches are used to develop ultrasensitive sensors that include employing different signal amplification techniques that use nanoparticles with unique optical, photophysical, electrical, magnetic, catalytic, and other properties [87]. On the other hand, the use of nanoparticles is limited by the difficulty of fabrication [88]. In addition to the constraining size, cost, speed, and accuracy requirements, transferring sensing technologies from specialized laboratories into point-of-care devices faces another challenge. This lies in system integration for the different parts of a POC device [89]. Integration of subsystems for blood sampling, microfluidic processing, target recognition and signal transduction, signal measurement, and power supply imposes additional challenges on the choice and design of nano biosensors whose integration must be feasible within the overall scheme [90]. Challenges of individual subsystems include the need for low sample consumption, separation of target molecule(s), multiplexing, processing a milliliter-range blood sample at the micro scale, prolonged storage of bioreceptors and reagents, and a user-friendly compact overall design. These challenges must not be overlooked when designing the detection subsystem of the device as each biosensing technique imposes certain compatibility issues on the other subsystems. A major bottleneck appears at the very initial stage of blood sample collection [91] with concerns on the matrix effect (i.e., existence of several interfering molecules in the sample), the wide dynamic range of the analyte, the relatively low analyte concentration in the highly viscous background of whole blood, and the need to reduce the volume of the collected sample for subsequent processing. Addressing such challenges first necessitates separating the analyte from the background either by labeling or by means of spatial or regional separation techniques [92]. From a system integration perspective, the choice of the separation technique must not be independent of the biosensor design. Biosensors that rely on magnetic nanoparticles can use magnetic bead-based separation while optical biosensors can rely on labelling. Each separation technique adds limitations for the overall sensing device. Optical approaches require relatively bulky equipment with increasing complexity for multiplexing while spatial and regional separation present challenges in device fabrication and assay preparation.

Various microfluidic techniques have been proposed for whole blood separation. The unique features of microfluidics such as their miniaturized size and their special electro-fluidic and thermal characteristics allows their use in POC [93]. For instance, the Zweifach–Fung bifurication effect on branch points of microfluidic channels allow the rapid separation of blood, which travels into channels with higher flow rates, from plasma, which travels into lower flow rate channels [94]. Coupling bead-based capture approaches with microfluidic systems allow efficient separation of the analyte at low concentrations [95,96]. To further increase the manipulability of the beads, magnetic and electrokinetic forces can be utilized. Specifically, electrokinetics, which result in a net electric force on a particle that is placed in a non-uniform electric field and possess certain electrical properties [93], presents a suitable alternative for integration with electrochemical biosensors that share a similar electrode interface [97,98]. Such integration of advanced microfluidic systems with biosensors is crucial for the development of POC devices that are fully automated and require no technical intervention from an expert. In addition to the discussed challenges of biosensors on the target recognition and signal transduction levels, challenges in the earlier stages of sample preparation must be taken into account in the general design and requirements of biosensors for POC devices since the choice of biosensors might impose constraining possibilities on other stages. Designing each component of the device as part of a complete system rather than as a separate entity is a key step towards achieving feasible POCT. While lots of work has been done for the separate optimization of the target recognition and signal transduction components (e.g., increasing the sensitivity, decreasing the LoD, or decreasing the response time), there has been limited efforts for understanding the interplay between all components of a POC device. Such interplay can give rise to potential compatibility issues when choosing a certain design for one of the subsystems.

Recently, paper-based sample pre-treatment technologies are gaining increased interest in POCT. These low-cost, rapid, easy-to-use, and eco-friendly techniques can tackle the sample pre-treatment steps of collection and storage, separation, extraction, and concentration offering great potential for low-resource-settings POCT [99]. However, further research is required to overcome some of their challenges and reach a fully-automated paper-based platforms [99]. To this end, not only the sample preparation is important, but also the readout convenience, which is being recently investigated in the scope of smartphone-based POCT. With a rich set of built-in sensors, display, memory, connectivity options, and compatible add-on devices, smartphones offer great capabilities for data collection, storage, analysis, display, and transmission for in vivo and in vitro tests [100]. Work in [101] presents an example of a custom-designed hand-held and cost-effective smartphone-based system that is able to match the performance of a conventional Food and Drug Administration (FDA)-approved ELISA reader. The use of smartphones for readout and analysis of physiological indexes has expanded in POCT diagnostics for CVDs; however, there is still a need for regularization and standardization of smartphones in POCT due to the non-standard and unstable features among the variety of available mobile phones [102].

### Environmental Factors

In contrast to typical controlled-laboratory testing, POCT devices that are intended for field applications can be affected by several environmental factors like temperature and relative humidity. Accumulating evidence has shown significant effects of such environmental factors on bio-molecular interactions, antigen-antibody interactions, and fluid wicking rates in paper-based assays [103]. These can influence assay readout and consequently result in a decrease of sensitivity. Therefore, it is necessary to monitor and control such environmental factors to maintain optimal operating conditions for enhanced sensitivity. In [103], authors developed a relatively small and portable temperature-humidity control device coupled with smartphone signal detection to provide optimal environmental requirements in lateral-flaw assay (LFA) POCT.

## 5. Conclusions

Heart failure epidemiology reveals high mortality and morbidity rates regardless of socio-economic class, gender, and age group, mainly owing to deficiencies in the existing diagnostic and disease management techniques. In order to tackle this public health issue, it became fundamental to adopt a holistic approach in optimizing diagnostics and therapy, by accounting for disease pathology at the molecular level and reducing laboratory testing time. This allowed for the dependence on cardiac biomarkers, most importantly BNP, in orienting clinical decisions and imposed a pressing need to revolutionize biosensing for rapid point-of-care applications in the emergency room. This review presented the latest trends in BNP sensing using both optical and electrochemical transducers. For these biosensors to replace the more complex and time-consuming conventional laboratory tests, several areas of potential shortcomings are to be addressed. This includes further investigation of multiplexing capabilities to increase the diagnostic accuracy by reducing false positive rates and maintaining high sensitivity for current BNP biosensing techniques. Another important area for improvement is on the device-level where easy-to-use BNP biosensors require the miniaturization of hardware components, which eventually requires more innovative sample collection, reagent delivery, and power supply units. In this context, the joint study of microfluidic channeling and biosensing techniques might lead to smoother integration and easier design processes. Current efforts in the field are promising, and many of the biosensors presented in this review have shown outstanding sensitivity and applicability in whole blood samples, which forms a solid basis for future in vivo BNP monitoring. Finally, the auspicious performance of the immunosensors as indicated by the sensitivity, detection limit, and reproducibility gives hope in translating similar work to clinical settings.

## Figures and Tables

**Table 1:** Comparison of promising cardiac biomarkers.

Cardiac Biomarker	Pathophysiological Model	Cut-off Value	Diagnosis	Prognosis	Therapy	Cardio-Specific
BNP	Cardiac Myocyte Stress	400 pg/mL	+	+	+	Yes
NT-proBNP	Cardiac Myocyte Stress	0.25–2 ng/mL	+	+	+	Yes
MR-proANP	Cardiac Myocyte Stress	120 pmol/L	+	+	?	Yes
Troponins	Myocyte Injury and Necrosis	0.01–0.1 ng/mL	+	+	?	Yes
Copeptin	Neurohormonal Activation	-	-	+	-	No
Myeloperoxidase	Oxidative Stress	350 ng/mL	-	+	?	No
NGAL	Renal Dysfunction	-	-	+	-	No
C-Reactive Protein	Inflammatory	$3\cdot 10^{3}$ ng/mL	-	+	?	No
Tumor necrosis factor	Inflammatory	0.0036 ng/mL	-	+	?	No
sST2	Adverse Cardiac Remodeling: cardiac stress, inflammation, and fibrosis	35 ng/mL	-	+	?	No

**Table 2:** Review of optical and electrochemical biosensors for BNP detection.

Ref	Technique	BNP Type	Stability	Reproducibility	Sensitivity	Selectivity	Sample Used	Incubation (min)	Response Time	Functionalization	Surface Characterization
[58]	Fluorescence-based	NT-pro BNP	-	<10% intra/inter assay variability	Linear Range: 200–26,000 pg/mL Limit of Detection: 47 pg/mL	-	Human serum, plasma, and whole blood	-	10 min	Covalent bonding (EDC/ NHS)	-
[59]	Optical-intensity based	BNP	-	-	Limit of Detection: 10 pg/mL	-	Whole blood	-	-	Biotinylated anti-BNP Anti-bodies binded to streptavidin-coated MMPs	UV-vis spectro-photometry
[60]	Electrochemi-luminiscence-based	NT-pro BNP	±1.9%/12 cycles	3.48% intra-assay precision 2.73% inter-assay precision	Linear Range: 0.1 pg/mL–25 ng/mL Limit of Detection: 0.05 pg/mL	Higher response than that to AFP, Col IV, and PSA	Undiluted human serum	45	-	Sandwich immunoassay: glassy carbon electrodes with NT-pro BNP Ab1 & PTC-Lu/PdCu@SWCNHs with Ab2	SEM, XPS, and CV
[63]	SPR-based	BNP	-	-	Linear Range: 5 pg/mL–100 ng/mL Limit of Detection: 5 pg/mL	-	Human serum	30	Real-time	BNP-modified Micro-channel by EDC/NHS covalent bonding	-
[64]	SPR-based	BNP	-	-	Linear Range: 1 aM–500 nM Limit of Detection: 1 aM	Non-specific adsorption	Undiluted human serum	60	Real-time	Sandwich immunoassay: EDC/NHS functionalizaton of BNP aptamers and 50 nm nanocubes with secondary anti-BNP	UV-vis spectroscopy and TEM
[65]	SPR-based	BNP	-	-	Linear Range: 10–100 ng/mL Limit of Detection: 25 pg/mL	Non-specific adsorption in human plasma	BNP buffer solution + human plasma	-	Real-time	Sandwich immunoassay with EDC/NHS covalent bonding of primary anti-BNP	-
[66]	SPR-based	BNP	-	-	Linear Range: 1 aM–500 nM Limit of Detection: 0.1 ng/mL	CRP, Myo, and cTnI	Human serum	10–15	-	Sandwich immunoassay	TEM + UV/vis spectro-photometry
[67]	Fluorescence-based	BNP	-	-	Linear Range: 26–260 pg/mL Limit of Detection: 26 pg/mL	-	-	5	-	Sandwich immunoassay Functionalization of quartz fiber with anti-BNP (EDC/NHS)	-
[71]	Fluorescence-based	NT-pro BNP	-	Less than 10% variation	Linear Range: 7–600 pg/mL Limit of Detection: 3.7 pg/mL	Low interference for hemoglobin, Bilirubin, intra-lipid, and biotin	Human plasma	11	-	Immobilized with oligonucleotides using 9G technology	-
[78]	Potentiometric	BNP	-	for 20 devices	Linear Range: 50–200 pg/mL Nonlinear Range: 50–1000 pg/mL	±5% for Myo, cTni, CK-MB	Serum	-	1 min	Covalent bond (EDC/NHS)	SEM, AFM
[79]	Potentiometric	BNP	Regenerated over one week and for 3 cycles	for 2 devices	Linear Range: 100 fM–1nM	<13% for BSA, D-Dimer, HAS	Whole Blood	30	10 s	Covalent bond (EDC/NHS)	SEM, TEM, EDS, XPS
[80]	Potentiometric	BNP	-	-	Linear Range: 0–1000 pg/mL	Spiked BNP concentrations in whole blood	Purified BNP + spiked BNP in whole blood+ whole blood	5	-	-	-
[81]	Amperometric	NT-pro BNP	-	-	Linear Range: 0.04–2.5 ng/mL Limit of Detection: 0.03 ng/mL	-	Human serum	16	-	Biotin-avidin interactions + ferrite permanent magnet for magnetic nanoparticle immobilization	-
[82]	Amperometric	NT-pro BNP	±2.3% /month ±2.1%/50 cycles	-	Linear Range: 0.005–1.67 ng/mL 1.67–4 ng/mL Limit of Detection: 0.003 ng/mL	<4.3% for BSA, healthy serum, dopamine, *v*/*v* Tween 20, and vitamin C	Serum + Whole Blood	18	-	Sandwich immunoassay via biotin-avidin interactions + ferrite permanent magnet for magnetic nanoparticle immobilization	TEM, AFM, XPS
[83]	Amperometric	BNP	Stable/25 days	4.7%–6.4% for 5 devices	Linear Range: 0.014 and 15 ng/mL Limit of Detection: 4 pg/mL	No significant difference with CRP, cTnI, cTNT, L(a), TNF alpha, IL-8, NTproBNP, and AXL	Serum	45	-	Aryl diazonium salt chemistry using 4-amino-thiopheno	AFM, SEM, EDX
[84]	Impedance-Based	BNP	-	for 3 devices	Linear Range: 1 ag/mL–10 ug/mL	No CRP cross-reactivity	Serum	15	Near real-time	Covalent bond between antibody-linkers and amine of thiol (DSP/DMSO)	Optical micrograph, SEM
[86]	Conductometric	BNP	-	-	Linear Range: 50 fg/mL–3 ng/mL	High confidence for BSA + others	Serum	-	-	Covalent bond (EDC/NHS)	SEM, Raman spectroscopy
